# Multiple infections with Omicron variants increase breadth and potency of Omicron-specific neutralizing antibodies

**DOI:** 10.1038/s41421-025-00800-5

**Published:** 2025-05-20

**Authors:** Lei You, Luning Zhang, Shengqun Ouyang, Bo Gao, Yanan Li, Jialu Li, Ningbo Wu, Hong Wang, Shiqi Sun, Jinfeng Li, Zi Yin, Ziyang Xu, Yao Chen, Yiwen Zhu, Shuangyan Zhang, Zhan Xu, Tianyu Zhang, Zhaoyuan Liu, Chuanxin Huang, Bin Li, Jieming Qu, Bing Su, Leng-Siew Yeap

**Affiliations:** 1 Shanghai Institute of Immunology and Department of Pulmonary and Critical Care Medicine, Center for Immune-Related Diseases at Shanghai Institute of Immunology, Ruijin Hospital, Institute of Respiratory Diseases, Shanghai Jiao Tong University School of Medicine, Shanghai, China; 2 Shanghai Key Laboratory of Emergency Prevention, Diagnosis and Treatment of Respiratory Infectious Diseases, Shanghai, China; 3 National Research Center for Translational Medicine at Shanghai, Shanghai, China

**Keywords:** Immunology, Mechanisms of disease

## Abstract

Despite high vaccination rates, highly evolved Omicron variants have caused widespread infections and, in some cases, recurrent infections in the human population. As the population continues to be threatened by new variants, it is critical to understand how the dynamic cross-reactive antibody response evolves and affects protection. Here, we longitudinally profiled neutralizing antibodies in individuals who experienced three Omicron waves in China over an 18-month period following the lifting of the COVID restriction. We found that individuals with BA.5/BF.7 and XBB dual infections had increased breadth and neutralizing potency of Omicron-specific antibodies compared to those with a BA.5/BF.7 single infection, and were thus more resistant to JN.1/XDV.1 infection in the third wave. During the second infection, a new imprint based on the previously infected variant was established, and the antibodies developed high cross-reactivity against the Omicron variants and less against vaccine-derived WT SARS-CoV-2. Our results suggest that the high titer and breadth of cross-reactive antibodies from multiple infections may be protective against future infection with Omicron variants such as JN.1, but may still be vulnerable to antigenically advanced subvariants such as KP.3.1.1 and XEC.

## Introduction

The severe acute respiratory syndrome coronavirus 2 (SARS-CoV-2) has continued to evolve since its emergence in 2019^1^. In China, the inactivated vaccine based on the original SARS-CoV-2 was introduced in January 2021^2^. Despite a high vaccination rate (92.5%) in the population^3^, the lifting of restrictions on COVID-19 in December 2022 was accompanied by nationwide infection with the Omicron variants BA.5/BF.7^4,5^. About six months later, around June 2023, a significant proportion of the population experienced a second infection with new Omicron variants similar to XBB.1.5. Compared to the BA.5/BF.7 wave, which affected the majority of the population when the restriction was lifted, the XBB wave appeared to be mild, as it gradually affected a smaller proportion of the population^4,6^. Since late 2023, a new Omicron variant, JN.1 and its subvariants, have continued to pose a threat, although the majority of the population has acquired hybrid immunity through vaccination and single or multiple Omicron infections. How the heterogeneity of immunity in the population provides protection against infection with the emerging Omicron variant remains unknown.

One of the factors that determine the level of protection against a new variant infection is the “recallability” of the immune memory and the degree of cross-reactivity and potency of the neutralizing antibodies shaped by previous exposure to SARS-CoV-2, either by vaccination and/or infection^7–11^. Studies by our group and others have shown that breakthrough infection with an Omicron variant, BA.1/BA.2, induces “recallability” or immune imprinting on the vaccinated SARS-CoV-2, resulting in dampened neutralizing antibody titers to BA.1/BA.2 variant^12,13^. Subsequent reports have shown that repeated breakthrough infections with multiple Omicron variants may override the immune imprint induced by vaccination based on the ancestral (hereafter referred to as WT) strain, as neutralizing antibodies after the second round of Omicron infection have decreased activities for the WT but increased activities for the first-infected Omicron variant^14–18^. Whether the new immune imprint changes the degree of cross-reactivity and the potency of the Omicron-specific neutralizing antibodies to protect against emerging Omicron variants remains to be investigated. A better understanding of the breadth of neutralization and the evolution of cross-reactive antibodies will aid in the assessment of protection against reinfection and will be critical for the rational design of future vaccines^19^.

In this study, we longitudinally profiled the neutralizing antibody titers of a total of 48 individuals who had experienced three waves of Omicron exposure, specifically the BA.5/BF.7 wave, the XBB wave, and the JN.1 wave, over an 18-month period beginning with the lifting of the COVID-19 restriction in China. We found that the level of antibody cross-reactivity generated by the previous infection(s) could predict whether an individual would be re-infected in the subsequent Omicron wave. Using antibody depletion assays, we further demonstrate that multiple Omicron infections can increase the breadth and potency of the antibody response to Omicron variants. These antibodies are largely cross-reactive against the Omicron variants, but less against the original SARS-CoV-2, and contribute to protection against reinfection with the antigenically drifting Omicron variants such as JN.1/XDV.1, but not KP.3.1.1. and XEC.

## Results

### The breadth and level of cross-reactive antibodies predict protection or reinfection

We conducted a longitudinal study to examine the breadth and cross-reactivity of the antibody response in individuals who have experienced multiple waves of Omicron exposure. Forty-eight participants were enrolled and followed up four times from January 11, 2023 to June 20, 2024 (Supplementary Fig. S1). Time 1 (T1) and Time 2 (T2) correspond to the period of the BA.5/BF.7 wave in January and February 2023, respectively, Time 3 (T3) corresponds to the period of the XBB.1.5 wave in July 2023, and Time 4 (T4) corresponds to the period of the JN.1 wave in June 2024. Forty-four of the participants had received at least two doses of inactivated SARS-CoV-2 vaccine, and 39 of them had received a booster dose of the same vaccine between 6 and 17 months before the study. A total of 141 serum samples were collected, including one to four samples from each participant, and 47 participants provided at least two samples. We performed pseudovirus neutralization assays against Omicron variants circulating in China during the study period, BA.5, BF.7, XBB.1.5, JN.1, and XDV.1^4^, as well as the original SARS-CoV-2 strain (WT), the Omicron variant BQ.1.1, and SARS-CoV, and the recently circulating JN.1 subvariants KP.3, KP.3.1.1, and XEC. Individuals who received 3 doses of inactivated virus vaccine have little or no nAb against BA.5^20^, and we consider individuals with BA.5/BF.7 nAb titers higher than 1:200 at T1 to be infected with BA.5/BF.7. At T3, the same participants who had a fourfold or greater increase in XBB.1.5 nAb compared to T1/T2 were considered infected with XBB. Based on these criteria, the participants were divided into two main groups: the first group was infected once with BA.5/BF.7 in wave 1 but not with XBB variants in wave 2 (*n* = 10); the second group was infected twice, with BA5/BF.7 and XBB variants in the two waves (*n* = 12). The demographics and vaccination history of these participants are shown in Supplementary Table S1. Participants whose antibody profiles did not fit into these two categories, e.g., those who escaped infection in both waves (*n* = 1), or those who were infected in the later wave but not in the earlier wave (*n* = 4), or those who did not have samples collected at both T1 and T3 (*n* = 19), or those who received less than two doses of SARS-CoV-2 vaccines (*n* = 2), were not analyzed.

**Fig. 1:**
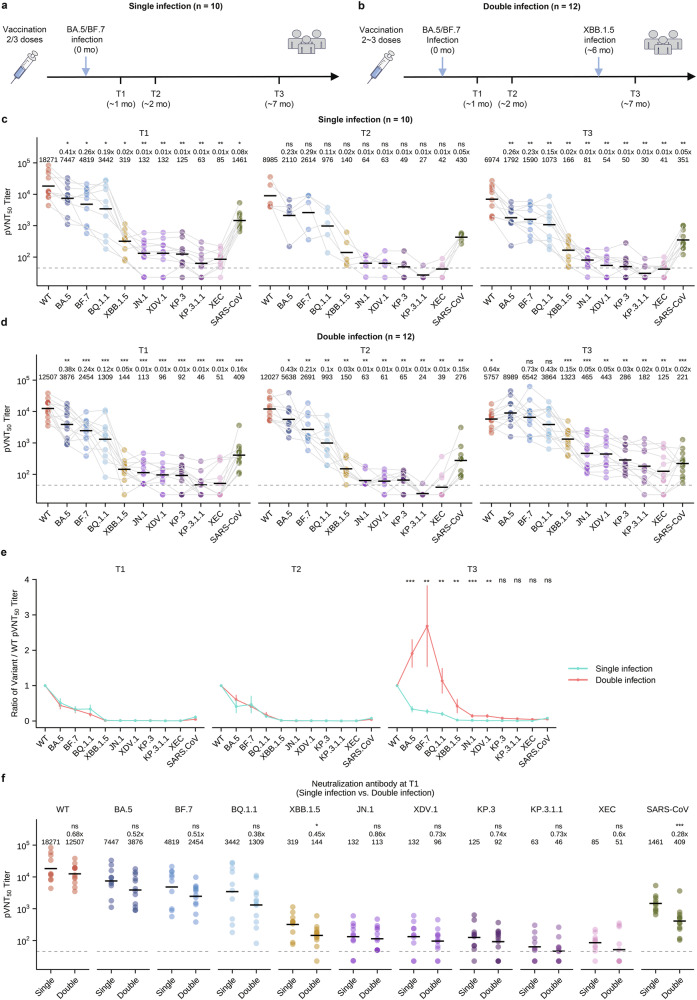
Cross-reactive antibodies predict protection or reinfection. **a**, **b** Schematic of blood collection in participants with single BA.5/BF.7 infection (**a**) or double BA.5/BF.7 and XBB infection (**b**). **c**, **d** Pseudovirus neutralization assays were performed using serum samples collected at the times indicated in **a** (*n* = 10 at T1 and T3, *n* = 5 at T2) and **b** (*n* = 12 at T1 and T3, *n* = 10 at T2) against WT SARS-CoV-2, BA.5, BF.7, BQ.1.1, XBB.1.5, JN.1, XDV.1, KP.3, KP.3.1.1, XEC variant strains and SARS-CoV pseudoviruses. pVNT_50_ values are reciprocal serum dilutions at which the relative light units were reduced by 50%. Each dot represents data from a serum sample, and dots for the same sample are connected with grey lines. **e** The ratio of pVNT_50_ titers against the indicated variants to titer against WT in individuals with the indicated history of infection. **f** Parallel comparisons of pVNT_50_ titers against the indicated variants at T1 between the single and double infection groups. Dashed lines indicate the limit of detection (pVNT_50_ = 45). Geometric mean titers (GMTs) are labeled as black lines and shown above each column (**c**, **d**, **f**), with fold-changes and significance compared with the variant having the highest titer (**c**, **d**) or with the single infection group (**f**) shown on the top. *P < 0.05, **P < 0.01, ***P < 0.001; ns, not significant.

We first compared the neutralizing antibody profiles of the single infection group (infected with BA.5/BF.7 at T1) and the double infection group (infected with BA.5/BF.7 at T1 and XBB variants at T3) between T1 and T3 (Fig. 1a, b). The single infection group shows a gradual decrease in the neutralizing antibodies against all the variants tested, while the double infection group shows a significant increase in antibodies against XBB.1.5, JN.1, XDV.1, KP.3, and KP.3.1.1, and a slight increase in antibodies against BA.5, BF.7, BQ.1.1, and XEC, but not in WT and SARS-CoV neutralizing antibodies, from T1 to T3 (Supplementary Fig. S2). These profiles indicate that, first, SARS-CoV-2 and Omicron variant neutralizing antibodies decrease over time in the absence of reinfection; second, Omicron neutralizing antibodies, but not WT and SARS-CoV neutralizing antibodies, are increased after a second Omicron variant reinfection, thereby improving the breadth of Omicron neutralization; and third, neutralizing antibodies against future strains, JN.1 and its subvariants, were significantly increased after XBB reinfection, indicating potential protection against future strains in the double infection group. When the profiles were plotted to show the levels of the different variant neutralizing antibody titers at the three time points, we observed that neutralizing antibody titers against WT were the highest at all the time points in both groups except T3 in the double infection group, where neutralizing antibody titers against BA.5 and BF.7 were the highest (Fig. 1c, d). These results suggest that the initial breakthrough infection with BA.5/BF.7 recalled memory established by WT SARS-CoV-2 vaccination and that imprint lasted for at least 6 months, whereas a reinfection with XBB presented a new imprint established by the variant that caused the first infection, BA.5/BF.7. This notion is also supported by the observation that the ratio of BA.5 and BF.7 to the WT neutralizing antibody titer doubles at T3 in the double infection group (Fig. 1e).

To investigate whether the difference in neutralizing antibody levels was associated with XBB reinfection, we compared the neutralizing antibody titers of both groups at T1, before reinfection occurred. Neutralizing antibody titers for all variants tested were slightly lower, but not statistically significant, in the double infection group compared to the single infection group, except for XBB.1.5, which is significantly lower in the double infection group (Fig. 1f). However, the receptor binding domain (RBD)-binding antibodies for all variants tested did not demonstrate statistically significant differences between the double infection group and the single infection group (Supplementary Fig. S3). This result suggests that the lower levels of XBB.1.5 cross-neutralizing antibodies induced during BA.5/BF.7 infection may be associated with XBB reinfection in individuals in the double infection group.

### BA.5/BF.7 and XBB infections increase the neutralizing potency of JN.1 antibodies

**Fig. 2:**
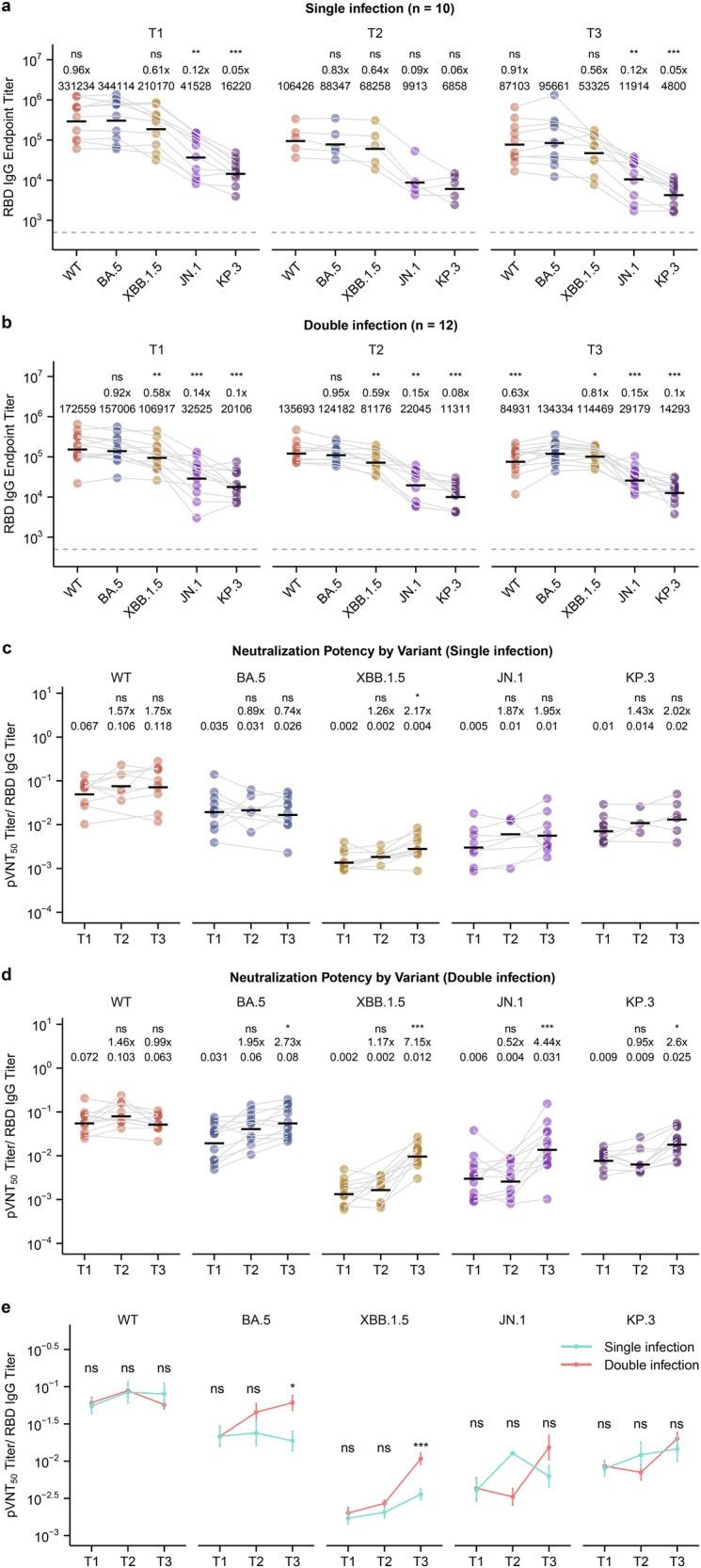
BA.5/BF.7 and XBB infections increase the neutralizing potency of JN.1 antibodies. **a**, **b** Spike RBD-specific IgG ELISAs were performed using sera collected at the indicated times against RBD proteins of SARS-CoV-2 WT, BA.5, XBB.1.5, JN.1, and KP.3 variants. Dashed lines indicate the limit of detection (IgG titer = 500). GMTs are labeled as black lines and shown above each column, with fold-changes and significance compared with the variant having the highest titer shown on the top. **c**, **d** Neutralization potency was calculated by dividing the pVNT_50_ values by the RBD IgG endpoint titers. Mean values are labeled as black lines and shown above each column, with fold-changes and significance compared with T1 shown on the top. **e** Comparison of the neutralization potency between single infection group and double infection group. Mean values are labeled as dots, with significance shown on the top. *P < 0.05, **P < 0.01, ***P < 0.001; ns, not significant.

We then asked whether the neutralizing potency of Omicron antibodies changed over time and with reinfection. Neutralization potency is defined as the neutralizing ability of a given amount of binding antibodies and is calculated as the neutralization antibody titer divided by the binding antibodies. Since most SARS-CoV-2 and Omicron neutralizing antibodies target the RBD^21^, we first performed ELISA experiments to measure the levels of RBD-binding IgG antibodies. As expected, WT and the tested Omicron RBD-binding antibodies decreased over time in the single infection group, whereas only WT but not Omicron RBD-binding antibodies decreased over time in the double infection group (Supplementary Fig. S4). The RBD-binding antibody profile also suggests that Omicron reinfection leads to a change in immune imprint from WT to BA.5, as the level of BA.5 RBD-binding antibody was the highest at T3 in the double infection group but not in the single infection group (Fig. 2a, b). When antibody neutralizing potency was analyzed, we observed that in the single infection group, only XBB.1.5, but not the other variants tested, had a significant increase in antibody neutralizing potency from T1 to T3 (Fig. 2c), suggesting affinity maturation in cross-neutralizing XBB.1.5 antibodies. Reinfection with XBB not only dramatically increased the neutralizing potency of XBB.1.5 antibodies, but also the neutralizing potency of BA.5, JN.1, and KP.3 antibodies (Fig. 2d). In addition, BA.5 and XBB.1.5 antibody neutralization potency at T3 was also higher in the double infection group than in the single infection group (Fig. 2e). Taken together, these results suggest that Omicron infection increases the breadth and neutralization potency of Omicron variant antibodies, including the future variant JN.1.

### Multiple infections of Omicron variants decrease antibodies that cross-bind to WT SARS-CoV-2

**Fig. 3:**
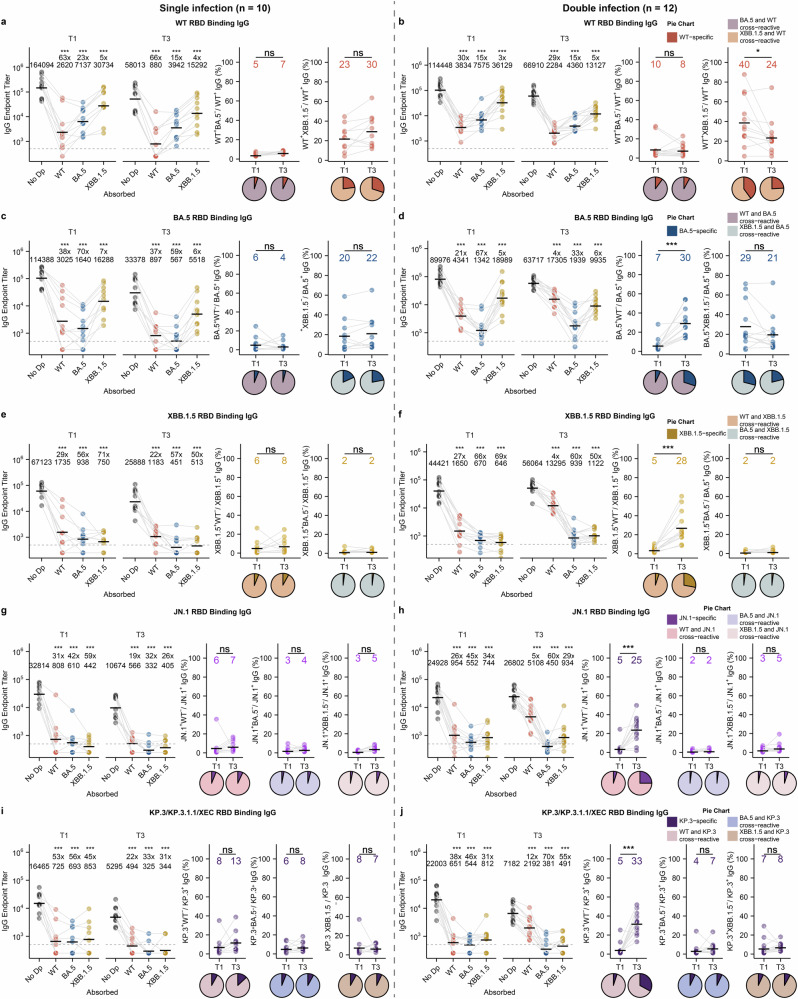
Multiple Omicron reinfection decreases antibodies that cross-bind to WT SARS-CoV-2. **a**–**j** Serum samples collected at T1 and T3 from individuals with single infection (**a**, **c**, **e**, **g**, **i**, *n* = 10) and double infection (**b**, **d**, **f**, **h**, **j**, *n* = 12) were absorbed with empty beads (No Dp) and beads coated with RBD proteins of WT, BA.5 or XBB variants. ELISA was then performed on the preabsorbed samples. The IgG endpoint titer in RBD-absorbed samples was compared with the titer in no Dp samples. Dashed lines indicate the limit of detection (IgG titer = 500); GMTs are labeled as black lines and shown above each column, with fold-changes and significance compared with the no Dp control shown on the top (left panels). The percentages of variant RBD-specific antibodies were compared for samples between T1 and T3 (means of each column are labeled as black lines and shown above), with pie charts illustrating the proportion of antigen-specific and cross-reactive IgG in each group (middle and right panels). *P < 0.05, **P < 0.01, ***P < 0.001; ns, not significant.

Next, we further explored how the antibody cross-reactivity changed in the single and double infection groups. We performed antibody depletion assays using beads coated with either SARS-CoV-2 WT, BA.5 or XBB RBD proteins. Antibodies that were bound to the specific antigen-coated beads were removed, and the remaining antibodies in the serum were used to test the binding and neutralizing activities against different variants (Supplementary Fig. S5). Antibodies specific to a particular antigen are considered to be efficiently removed if the level of the antibody binding titer is reduced by at least 90% after the depletion assay with the particular antigen. Antibody cross-reactivity between two variants is represented by the proportion of reduction in antibody binding titer to one variant after a depletion assay with an antigen of another variant. We first analyzed the antibody cross-reactivity between WT and other variants. In the single infection group, WT-specific antibody is efficiently removed by WT RBD beads (63-fold lower antibody titer than no depletion group), but less so by BA.5 or XBB RBD beads (23-fold and 5-fold lower than no depletion group, respectively) (Fig. 3a). The proportion of WT-specific antibodies to WT and BA.5 cross-reactive antibodies did not change from T1 to T3, and only about 5%–7% of the WT binding antibodies failed to cross-react with BA.5 (see Methods) (Fig. 3a, middle panel). The proportion of WT-specific antibodies to WT and XBB cross-reactive antibodies also did not change from T1 to T3, but the degree of antibody cross-reactivity of WT and BA.5 was higher than that of WT and XBB (Fig. 3a, right panel). In the double infection group, the level of WT and XBB, but not WT and BA.5, cross-reactive antibodies increased from T1 to T3, indicating that XBB infection activates antibodies cross-reactive between WT and XBB (Fig. 3b). Interestingly, the proportion of WT and XBB cross-reactive antibodies tended to be higher (although not significant) at T1 in the single infection group compared to the double infection group, suggesting that the higher level of XBB cross-reactive antibodies contributed to the protection against XBB infection (Fig. 3a, b).

Next, we analyzed the cross-reactivity of the antibodies between the different Omicron variants. Among the BA.5 binding antibodies, a large proportion cross-reacted with WT or XBB at T1, but the degree of cross-reactivity between BA.5 and WT was higher than that between BA.5 and XBB (Fig. 3c, middle and right panels). The proportion of cross-reactivity between BA.5 and WT or BA.5 and XBB remained unchanged from T1 to T3 in the single infection group. In contrast, the proportion of cross-reactivity between BA.5 and WT decreased, while the proportion of cross-reactivity between BA.5 and XBB increased from T1 to T3 in the double infection group (Fig. 3d, middle and right panels). This result suggests that XBB reinfection increases antibodies that are cross-reactive between BA.5 and XBB. Similarly, for XBB-binding antibodies, a large proportion cross-reacted with WT or BA.5 at T1 in both groups and did not change over time in the single infection group (Fig. 3e). However, in the double infection group, the proportion of XBB and WT cross-reactivity decreased from T1 to T3, while the degree of XBB and BA.5 cross-reactivity remained very high (98%), supporting the notion that XBB infection enhances antibodies that are cross-reactive between BA.5 and XBB (Fig. 3f). Similarly, a high proportion of JN.1 binding antibodies were cross-reactive with WT, BA.5 or XBB, and the proportion of cross-reactivity between JN.1 and WT decreased from T1 to T3 in the double infection group (Fig. 3g, h). Several JN.1 subvariants, including KP.3, KP.3.1.1, and XEC, have circulated worldwide since mid-2024 and they share the same spike RBD sequences^22^. Among the KP.3 RBD binding antibodies, a high proportion cross-bound with WT, BA.5, and XBB RBD, and the cross-reactivity with WT RBD decreased at T3 in the double infection group (Fig. 3i, j). Taken together, these results suggest that XBB reinfection reduces the proportion of antibodies that are cross-reactive with WT SARS-CoV-2.

### XBB reinfection elicits cross-neutralizing antibodies among Omicron variants including JN.1 and XEC

**Fig. 4:**
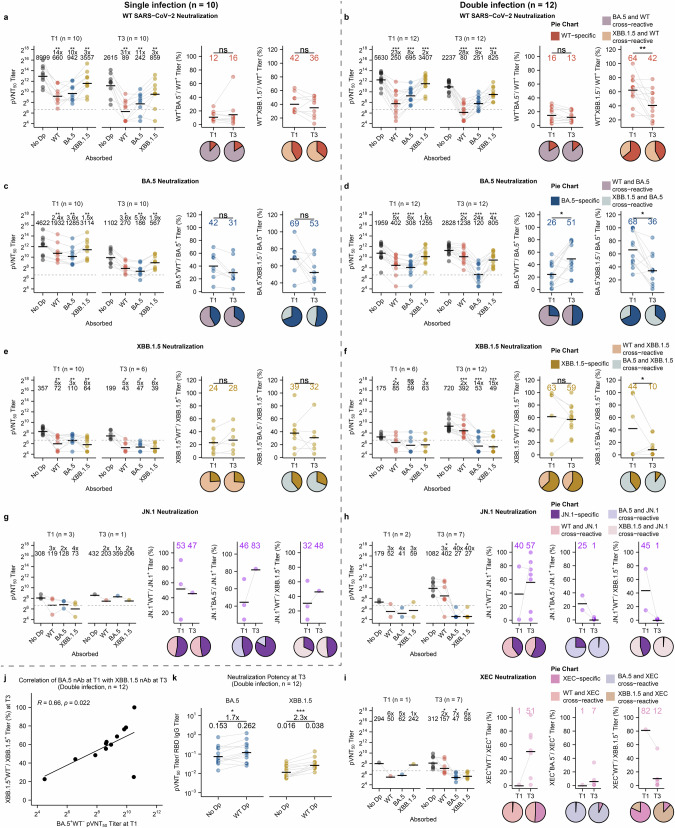
XBB reinfection elicits cross-neutralizing antibodies among Omicron variants including JN.1 and XEC. **a**–**i** Serum samples collected at T1 and T3 from individuals with single infection (**a**, **c**, **e**, **g**) and double infection (**b**, **d**, **f**, **h**, **i**) were absorbed with empty beads (No Dp) or beads coated with RBD proteins of WT, BA.5 or XBB variants, and then used for pseudovirus neutralization assays. Neutralization assays were performed at a starting dilution of 1:100 due to the limited samples available. Only samples with positive values (pVNT_50_ > 100) for each variant were considered for cross-reactivity testing, and the number of samples (*n*) used in the assay is indicated at the top. The neutralization titer in RBD-absorbed samples was compared with the titer in no Dp samples. Dashed lines indicate the limit of detection (pVNT_50_ = 100); GMTs are labeled as black lines and shown above each column, with fold-changes and significance compared with the no Dp control shown at the top (left panels). The percentages of variant-specific neutralizing antibodies were compared for samples between T1 and T3 (means of each column are labeled as black lines with the number shown at the top), with pie charts illustrating the proportion of variant-specific and cross-reactive neutralizing antibodies in each group (middle and right panels). **j** Correlation of BA.5-specific neutralization antibody titer that did not cross-react with WT at T1 with the proportion of XBB.1.5 neutralization antibody that did not cross-react with WT at T3. Spearman correlation test was performed. **k** Neutralization potency of BA.5 and XBB antibodies before and after depletion of antibodies that cross-react with WT at T3 in the double infection group. Means of each column are labeled as black lines and shown at the top, with fold-changes and significance compared with the no Dp control shown at the top. *P < 0.05, **P < 0.01, ***P < 0.001; ns, not significant.

We further performed the pseudovirus neutralization assay after the depletion assay to determine the fractions of neutralizing antibodies that are cross-reactive between different variants. Neutralizing antibody titers decreased by over 72% after depletion with RBD-coupled beads (Fig. 4a–f, left panels), indicating that RBD is the major target of neutralizing antibodies, consistent with previous studies^21^. For WT antibodies, only a small fraction failed to cross-neutralize with BA.5 and this fraction did not change over time in either the single or double infection groups (Fig. 4a, b, middle panels). However, the proportion of WT antibodies cross-neutralizing with XBB increased significantly from T1 to T3 in the double infection group, indicating that XBB reinfection induces WT and XBB cross-neutralizing antibodies (Fig. 4a, b, right panels).

Consistent with the antibody cross-binding results, reinfection with XBB caused a decrease in BA.5 and WT cross-neutralizing antibodies but an increase in BA.5 and XBB cross-neutralizing antibodies (Fig. 4c–f), indicating that XBB reinfection generates antibodies that are more cross-reactive with the previously infected variant than with the vaccinated SARS-CoV-2. XBB neutralizing antibody titers were low and decreased to below the detection limit (1:100) after depletion assay with all variant RBD beads in the single infection group (Fig. 4e), indicating that all XBB neutralizing antibodies cross-react with WT and BA.5. In the double infection group, XBB and BA.5 cross-neutralizing antibodies increased at T3, supporting the notion of increased cross-reactivity with a previously infected variant (Fig. 4f). Only a few individuals had neutralizing antibodies against JN.1 at T1 (Fig. 4g, h). In the double infection group, JN.1 cross-neutralizing antibodies were induced by XBB reinfection in 7 of the 12 participants, and these antibodies cross-reacted with both BA.5 and XBB.1.5, including 2 that also cross-reacted with WT (Fig. 4h). We also tested the cross-reactivity of neutralizing antibodies to XEC, a new subvariant of JN.1 that has been circulating worldwide since late 2024. In the double infection group, XEC neutralizing antibodies were induced at low titers in 7 participants at T3 and were highly cross-reactive with BA.5, of which 6 and 2 were also cross-reactive with XBB.1.5 and WT, respectively (Fig. 4i). Taken together, these results suggest that XBB reinfection induces cross-neutralizing antibodies between the Omicron variants, particularly against JN.1, which may be protective against JN.1 and, to a lesser extent, XEC infection.

We also determined which of the serological factors determines the development of a variant-specific antibody response after XBB infection. The results showed that the proportion of XBB.1.5-specific neutralization antibodies that did not cross-react with WT RBD correlated with BA.5-specific neutralization antibody titers at T1 (Fig. 4j; Supplementary Fig. S6), indicating that BA.5/BF.7 infection induces immune memory cross-reactive with XBB.1.5. We further analyzed the neutralization potency of variant-specific antibodies, and found that the neutralization potencies of BA.5- and XBB.1.5-specific antibodies that did not cross-react with WT RBD were significantly higher than the potencies of total BA.5 and XBB.1.5 antibodies (Fig. 4k). Taking together, these results indicate that XBB infection enhances the immune memory induced by BA.5 infection, but not the immune memory established by WT SARS-CoV-2 vaccination, and that the response is broadly cross-reactive between Omicron variants.

### Double infection group tend to be more resistant to JN.1 infection

**Fig. 5:**
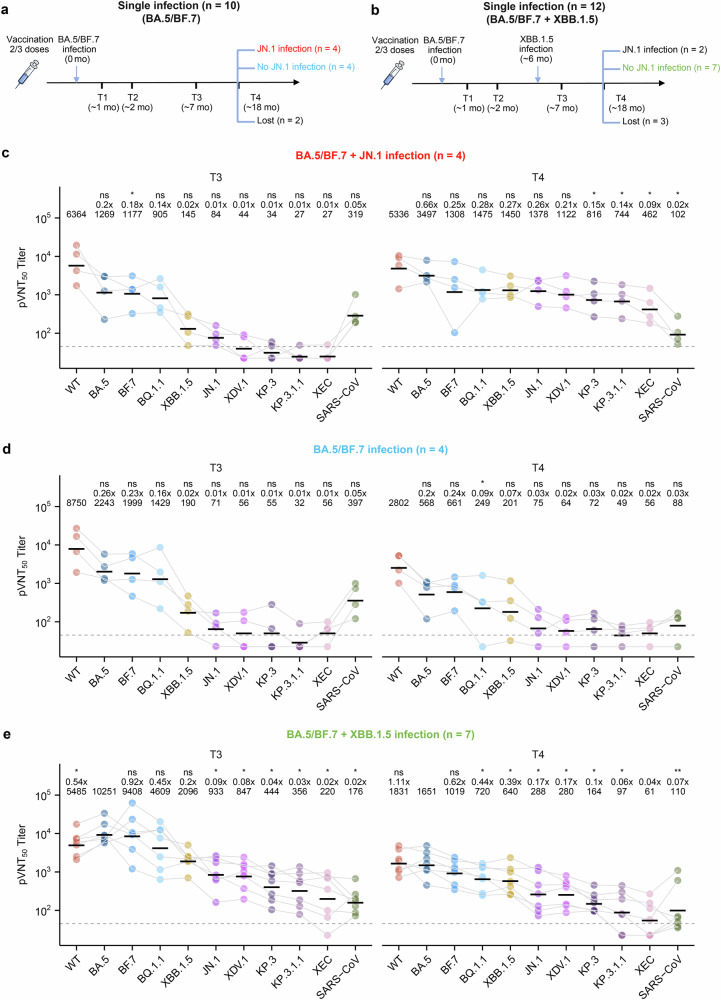
Double infection group tend to be more resistant to JN.1 infection. **a**, **b** Schematics of participants in the single infection group (**a**) and the double infection group (**b**) infected with JN.1. **c**–**e** Pseudovirus neutralization assays were performed using serum samples obtained at T3 and T4. Dashed lines indicate the limit of detection (pVNT_50_ = 45). GMTs are labeled as black lines and shown above each column, with fold-changes and significance compared with the variant having the highest titer shown at the top. *P < 0.05, **P < 0.01, ***P < 0.001; ns, not significant.

Based on our findings that dual infection with BA.5/BF.7 and XBB increased the breadth and potency of cross-neutralizing antibodies to Omicron variants, including JN.1, we predicted that individuals who had experienced dual infection would be more resistant to JN.1 infection. We performed a pseudovirus neutralization assay on serum samples from the same individuals in the two groups collected in June 2024 (T4), which is approximately 11 months after T3, and at a time when JN.1 and its subvariant XDV.1 were widely circulating in China^4^. Based on JN.1/XDV.1 neutralizing antibody titers at T3 and T4, 4 out of 8 (50%) individuals in the single infection group were infected with JN.1/XDV.1, whereas only 2 out of 9 (22%) individuals in the double infection group were infected with JN.1/XDV.1 (Fig. 5a, b). This result suggests that the individuals in the double infection group tend to be more resistant to JN.1 infection than individuals in the single infection group (*P* = 0.33, Fisher’s exact test). Similar to our observations that double infection (infected with BA.5/BF.7 and XBB) increased the breadth of cross-neutralizing antibodies, JN.1/XDV.1 infection significantly increased XBB cross-neutralizing antibody titers in individuals in the single infection group (Fig. 5c; Supplementary Fig. S7a). In other individuals not infected with JN.1/XDV.1 in the single infection group, the level of antibody against different variants continues to decrease. As the titers of the antibody against JN.1 and subvariants in this group were low and remained unchanged from T3 and T4, it is possible that individuals in the single infection group are more susceptible to infection of JN.1 and subvariants (Fig. 5d; Supplementary Fig. S7b). In contrast, the high levels of JN.1 and XDV.1 cross-neutralizing antibodies induced by double Omicron infections would tend to be protective against JN.1 and XDV.1 infection (Fig. 5e; Supplementary Fig. S7c). However, KP.3.1.1 and XEC neutralization antibody titers in the double infection group (BA.5/BF.7 and XBB) were low at T4, suggesting that these individuals may be vulnerable to infection by these JN.1 subvariants (Fig. 5e; Supplementary Fig. S7c).

Of the 48 participants enrolled in the cohort, 21 provided serum samples at both T3 and T4, 8 of whom were infected with JN.1/XDV.1. JN.1 and XDV.1 antibody titers at T3 were compared between individuals with and without JN.1/XDV.1 infection. The results showed that JN.1 and XDV.1 neutralization antibody titers were higher in those who were not infected by JN.1/XDV.1, and JN.1 binding antibodies also tended to be higher, indicating that cross-neutralization antibodies induced by previous Omicron infections are protective against JN.1/XDV.1 infection (Supplementary Fig. S8). As the JN.1 wave is still ongoing during the preparation of this manuscript, continued monitoring of the antibody response will be important to validate our findings. Taken together, our results demonstrate that repeated Omicron variant infection can increase the breadth of Omicron variant cross-neutralizing antibodies, which in turn protects against the infection of future new Omicron variants.

## Discussion

In this study, we analyzed the longitudinal cross-neutralizing antibody profiles of individuals who had experienced three waves of Omicron since the lifting of the COVID restriction in China. Participants enrolled in this study had relatively similar SARS-CoV-2 immune histories prior to the lifting of the restriction, as they were healthy adults who had received at least two doses of WT SARS-CoV-2 inactivated vaccine and had not been infected with SARS-CoV-2. After the first and the second Omicron waves, we found that the majority of participants could be divided into two groups: those who had experienced a single infection in the first Omicron wave but not the second Omicron wave, and those who had experienced double infections in both the first and second Omicron waves. After experiencing a BA.5/BF.7 breakthrough infection in the first wave, some individuals had higher XBB.1.5 cross-neutralizing antibodies than others with the same infection history and were protected against infection during the second wave of infection mainly by XBB. However, these individuals were more susceptible to the third wave infection by JN.1/XDV.1 than those in the double infection group. Our results also suggest that multiple Omicron infections may increase the breadth and potency of Omicron variants cross-neutralizing antibodies and reduce WT SARS-CoV-2 cross-neutralizing antibodies, thereby providing protection against a future Omicron variant. However, the individuals may still be vulnerable to a more antigenically advanced variant, such as KP.3.1.1 and XEC.

**Fig. 6:**
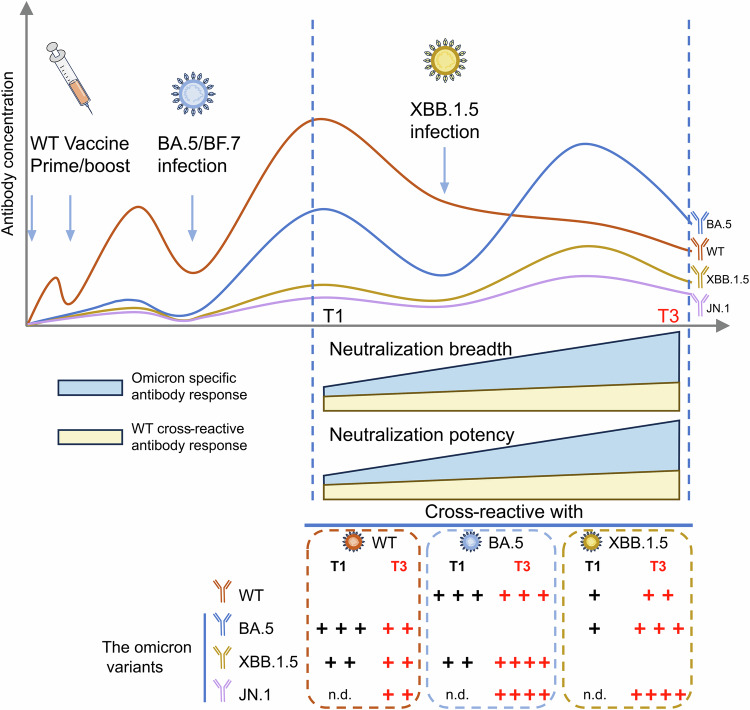
Impact of Omicron variants infections on the breadth and potency of SARS-CoV-2 neutralizing antibody response. Multiple infections with Omicron variants alleviate the immune imprinting induced by WT SARS-CoV-2 inactivated vaccine, establish a new imprint to Omicron variant, increase the breadth and potency of Omicron-specific antibodies, and induce a cross-reactive antibody response among Omicron variants, including JN.1. n.d., not detected.

Our study shows that the first Omicron variant infection by BA.5/BF.7 induces an immune imprint on the vaccinated SARS-CoV-2, but this effect is attenuated during a second Omicron variant infection by XBB as a new imprint. Although Omicron variant cross-reactive antibodies are increased during double infection, we show that the antibodies are still cross-reactive with WT, indicating that the immune memory induced by WT SARS-CoV-2 vaccination still contributes to the response induced by repeated Omicron infections. Therefore, the cross-neutralizing antibody response derived from the memory of WT SARS-CoV-2 vaccination is beneficial and contributes to protection against XBB reinfection. During the second infection, the elicited antibodies tend to cross-react more with the previously infected variant than with WT, probably due to the epitope masking effect of the strong WT neutralizing antibody response during the first Omicron infection^23^. Our study provides an overview of the dynamics and composition of the antibody response in a real-world Omicron infection (Fig. 6), which will facilitate the design of updated vaccines and the prevention of future outbreaks.

In contrast to the alleviation of WT SARS-CoV-2 inactivated vaccine-induced immune imprinting by infection with Omicron variants, WT SARS-CoV-2 mRNA vaccines have been reported to induce persistent immune imprinting in humans, even in subjects with repeated exposure to Omicron antigens, either by infection or vaccination^24–28^. Together with our findings, these data suggest that the imprinting effect is not only determined by the antigenically similar antigens but is also related to the vaccine platform and the type of exposure. Nevertheless, inactivated SARS-CoV-2 vaccines have been used worldwide^29^, and our observations would have broad implications. In addition, our data suggest a new imprinting towards the Omicron variant BA.5, which shapes the immune response of subsequent XBB infection. How this imprinting would change with further infections needs further investigation.

JN.1 has been the dominant Omicron variant circulating worldwide since its emergence in late 2023. In addition, JN.1 continues to evolve, and several descendant subvariants have emerged since mid-2024, with XEC being the most recent. Although double infections with BA.5/BF.7 and XBB variants induce antibodies that are cross-reactive among Omicron variants, including JN.1 and XEC, and may protect against JN.1 infection, XEC cross-neutralizing antibodies decline to low levels one year after XBB reinfection. In contrast, XEC neutralizing antibodies are high in individuals infected with BA.5/BF.7 and JN.1/XDV.1. Similarly, a recent study reported that JN.1 infection elicits a superior antibody response against its subvariants compared to XBB^30^. These data support the use of a JN.1 lineage variant in COVID-19 vaccines, in line with the WHO recommendation in December 2024^31^. Given the rapid evolution of SARS-CoV-2 and the decline in cross-neutralizing antibodies, our findings also support ongoing monitoring of SARS-CoV-2 variants and human immune responses and revaccination in high-risk populations^32^.

There are still imitations of the study. The first one is the small sample size. Nevertheless, our data have demonstrated the differences in antibody kinetics and cross-reactivity between the single and double infection groups, supporting our conclusions. Secondly, the experiments were limited to serum antibodies. Mucosal IgA has been shown to be critical for the prevention of Omicron breakthrough infections^33,34^; however, we were unable to obtain samples for mucosal antibody testing. Further studies should be conducted to analyze the cross-reactivity of mucosal IgA antibodies among SARS-CoV-2 variants. Although we only analyzed the binding and neutralizing antibody titers with pseudovirus assays and depletion assays, our conclusion remains valid as antibodies are one of the major component of the immune response that confers protection against invading viruses^35^. Future studies of cellular immunity, including B, T, and innate immune cells, will further elucidate the mechanisms underlying immune heterogeneity in the population.

## Materials and Methods

### Participants and samples

We recruited and followed 48 participants according to the study protocol performed in accordance with the Declaration of Helsinki and approved by the Ethics Committee of Ruijin Hospital (2021-73 and KY2024-58-A). Written informed consent was obtained from all participants. Blood samples were collected on January 11, 2023, February 15, 2023, July 4, 2023, and June 20, 2024 (referred to as T1, T2, T3, and T4, respectively). Sera were separated by centrifugation at 2000× *g* for 5 min at room temperature and stored in aliquots at –80 °C until use. Demographic information and SARS-CoV-2 vaccination history were collected from each participant and are listed in Supplementary Table S2.

### Pseudovirus neutralization assay

HEK293T cells were transfected with plasmids encoding SARS-CoV-2 spikes using Polyethylenimine (Polyscience). One day after transfection, the cells were infected with VSV-G pseudotyped ΔG-luciferase (G*ΔG-luciferase, Kerafast) at a multiplicity of infection of 5. Four hours after infection, the VSV-G pseudotyped ΔG-luciferase was removed. The cells were washed three times with phosphate-buffered saline and anti-VSV-G antibody was added. The cells were then maintained in fresh complete DMEM for two days. Cell culture supernatants containing pseudoviruses were collected and stored in aliquots at –80 °C until use.

Prior to neutralization assays, pseudoviruses were titrated using BHK21-ACE2 cells. Before testing, serum samples were inactivated at 56 °C for 30 min. 50 μL of diluted pseudoviruses were incubated with 50 μL of serial dilutions of sera in triplicate for 1 h at 37 °C. BHK21-ACE2 cells (2 × 10^4^ cells per well) were added. Virus control and cell control wells were included in each plate. Plates were then incubated at 37 °C for 20–24 h. Luminescence was measured using a Luciferase Assay System (Beyotime). NT_50_ was defined as the dilution at which the relative light units were reduced by 50% compared to the virus control wells after subtraction of the background in the cell control wells. The NT_50_ values were calculated using non-linear regression in GraphPad Prism.

### Enzyme-linked immunosorbent assay (ELISA)

Clear, flat-bottomed, hinged Binding 96-well plates (Costar, 42592) were coated with 2 µg/mL of recombinant protein (Supplementary Table S3) overnight at 4 °C. The next day, the plates were washed three times with PBS containing 0.1% Tween 20 (PBS-T) and blocked for 1 h with 300 µL blocking buffer (PBS-T supplemented with 2% BSA). The plates were then washed and 50 μL of serial dilutions of sera were added. After 2 h of incubation, the plates were washed 5 times with PBS-T and 50 µL of goat anti-human IgG HRP conjugate (Proteintech, SA00001-17) was added to each well and incubated for 1 h. The plates were then washed, and 50 µL of TMB substrate (Thermofisher, 00-4201-56) was added to each well. Plates were incubated in the dark for 12 min and the reaction was stopped by the addition of 1 M sulfuric acid stop solution. Plates were read immediately at OD_450_ nm.

### Antigen-specific antibody depletion

Biotinylated spike RBD proteins (Supplementary Table S3) were coupled to Magnetic Beads™ Streptavidin (ACROBiosystems, MBS-K002) at a ratio of 15 µg protein to 1 mg beads. The mixture of protein and beads was incubated for 1 h with rocking. Unbound antigen was removed and the beads were washed 3 times with PBS-T. For absorption, sera were diluted 1:10 with PBS and then incubated with empty beads (no Dp) or beads coated with RBD proteins for 1 h. Non-absorbed fractions were then separated by magnet and used for neutralization assays and ELISA. The amount of RBD protein required for complete depletion of IgG in serum samples was optimized and 0.05 μg RBD protein was sufficient to deplete IgG in 1 μL serum (depletion efficiency > 96% for neutralizing antibodies, and > 99% for RBD-binding antibodies) (Supplementary Fig. S5). An excess of RBD proteins at 0.1 μg per μL serum sample was used for the assay.

### Statistical analyses

The Wilcoxon matched-pairs signed rank test was used to compare neutralization and binding antibody titers against different variants and for samples absorbed with different antigens. The Mann-Whitney test was used to compare neutralization and binding antibody titers for samples at different time points. Spearman’s correlation was used to test the correlations with XBB or BA.5 neutralization antibodies at T1, and with XBB neutralization antibodies at T3. All statistical analyses were performed using GraphPad Prism (version 10.1.2).

## Supplementary information

Supplementary Information
