# Supplementary material for: Multiple infections with Omicron variants increase breadth and potency of Omicron-specific neutralizing antibodies
Source: Cell Discov. 2025 May 20;11:49. doi: 10.1038/s41421-025-00800-5 (PMC12089387; doi:10.1038/s41421-025-00800-5)
Supplement: Supplementary file 1 — Supplementary Information [file 41421_2025_800_MOESM1_ESM.pdf]

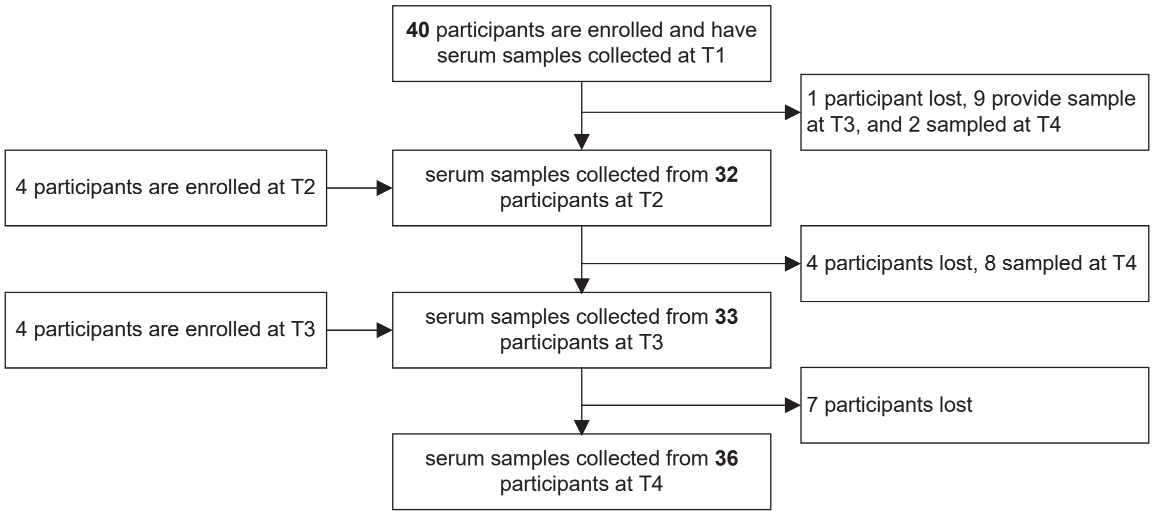

Supplementary Fig. S1 Flowchart of participant enrollment and sample collection.

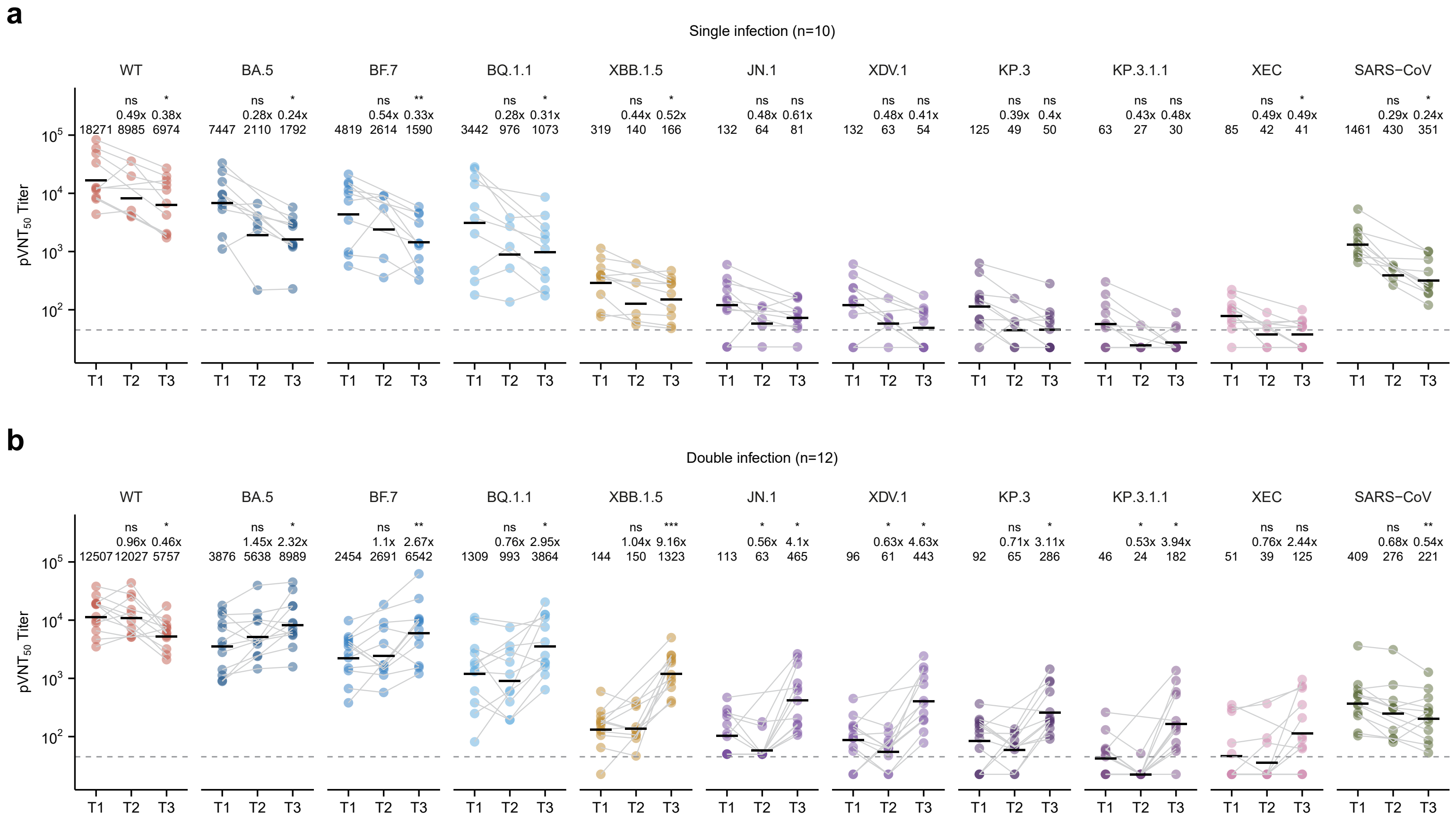

Supplementary Fig. S2 Dynamics of neutralization antibodies against SARS-CoV-2 variants in individuals with single and double Omicron infections.
Dashed lines indicate the limit of detection (pVNT50 = 45). Geometric mean titers (GMT) are labeled as black lines and shown above each column with fold-changes and significance compared with titer at T1 shown on the top. * $P < 0.05$, ** $P < 0.01$, *** $P < 0.001$; ns, not significant.

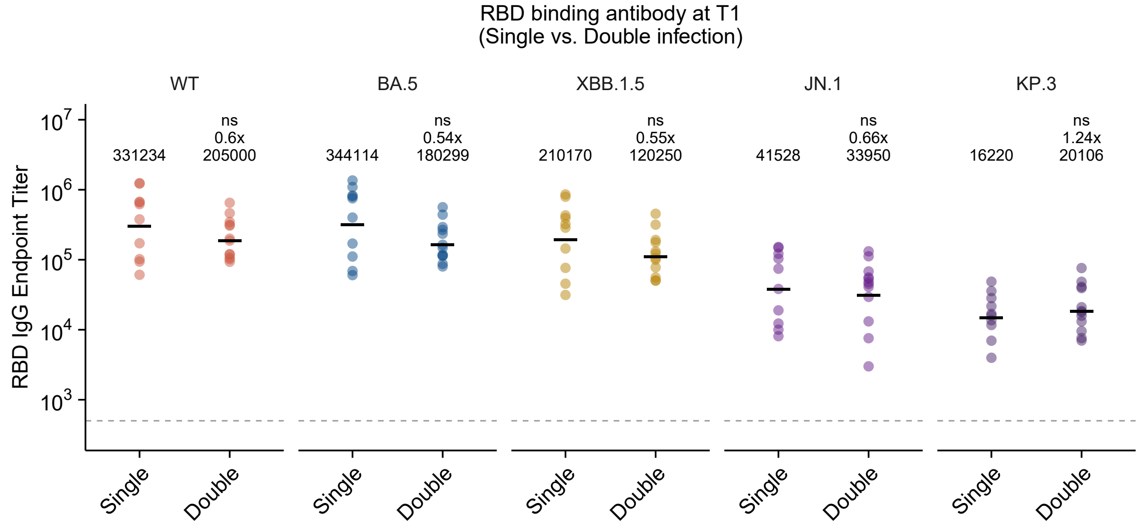

Supplementary Fig. S3 Comparisons of the indicated RBD IgG endpoint titers collected at T1 between the single infection group and the double infection group.

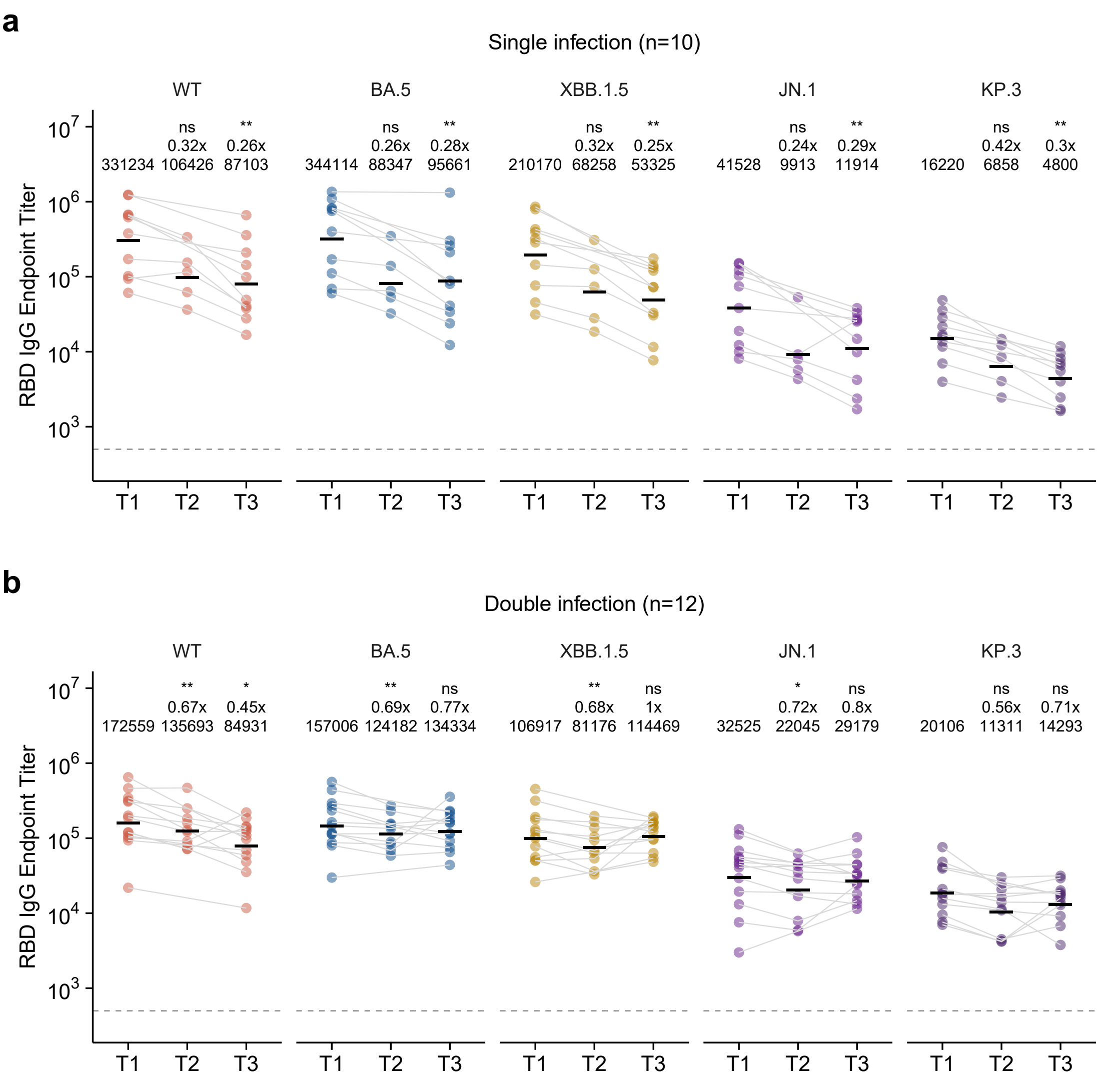

Supplementary Fig. S4 Dynamics of Spike RBD binding antibodies against SARS-CoV-2 variants in individuals with single and double Omicron infections.
Dashed lines indicate the limit of detection (IgG titer = 500). Geometric mean titers are labeled as black lines and shown above each column with fold-changes and significance compared with titer at T1 shown on the top. * $P < 0.05$, ** $P < 0.01$, *** $P < 0.001$; ns, not significant.

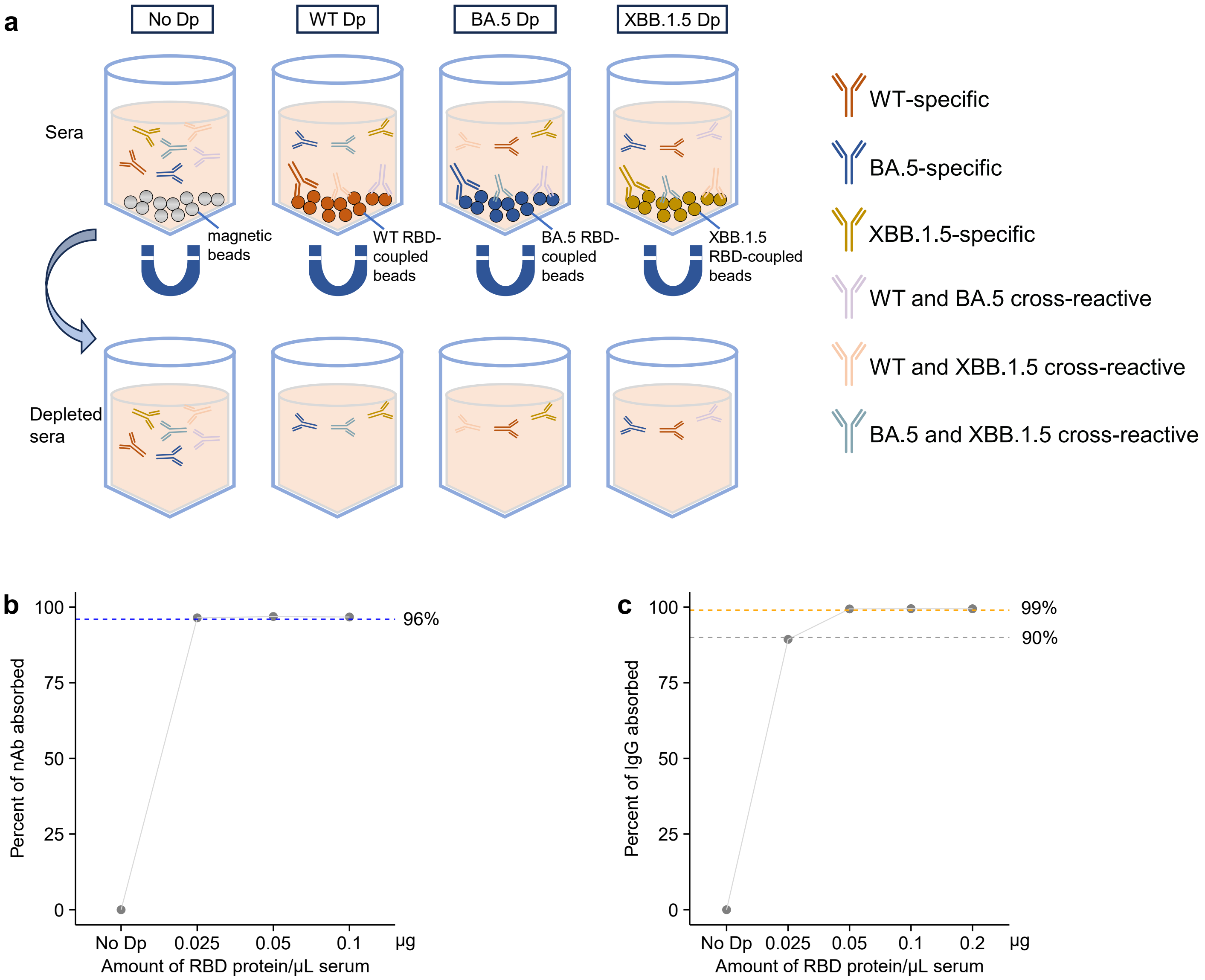

Supplementary Fig. S5 Antigen specific antibody depletion assay.
(a) Workflow of the antibody depletion assay.
(b, c) A serum sample with the highest WT SARS-CoV-2 neutralizing antibody titer was absorbed with a series of different doses of WT RBD proteins. The non-absorbed fractions were then used for neutralization assay and ELISA. Percentages of WT SARS-CoV-2 neutralizing antibody (b) and WT SARS-CoV-2 RBD binding antibody (c) absorbed compared to no depletion control are calculated.

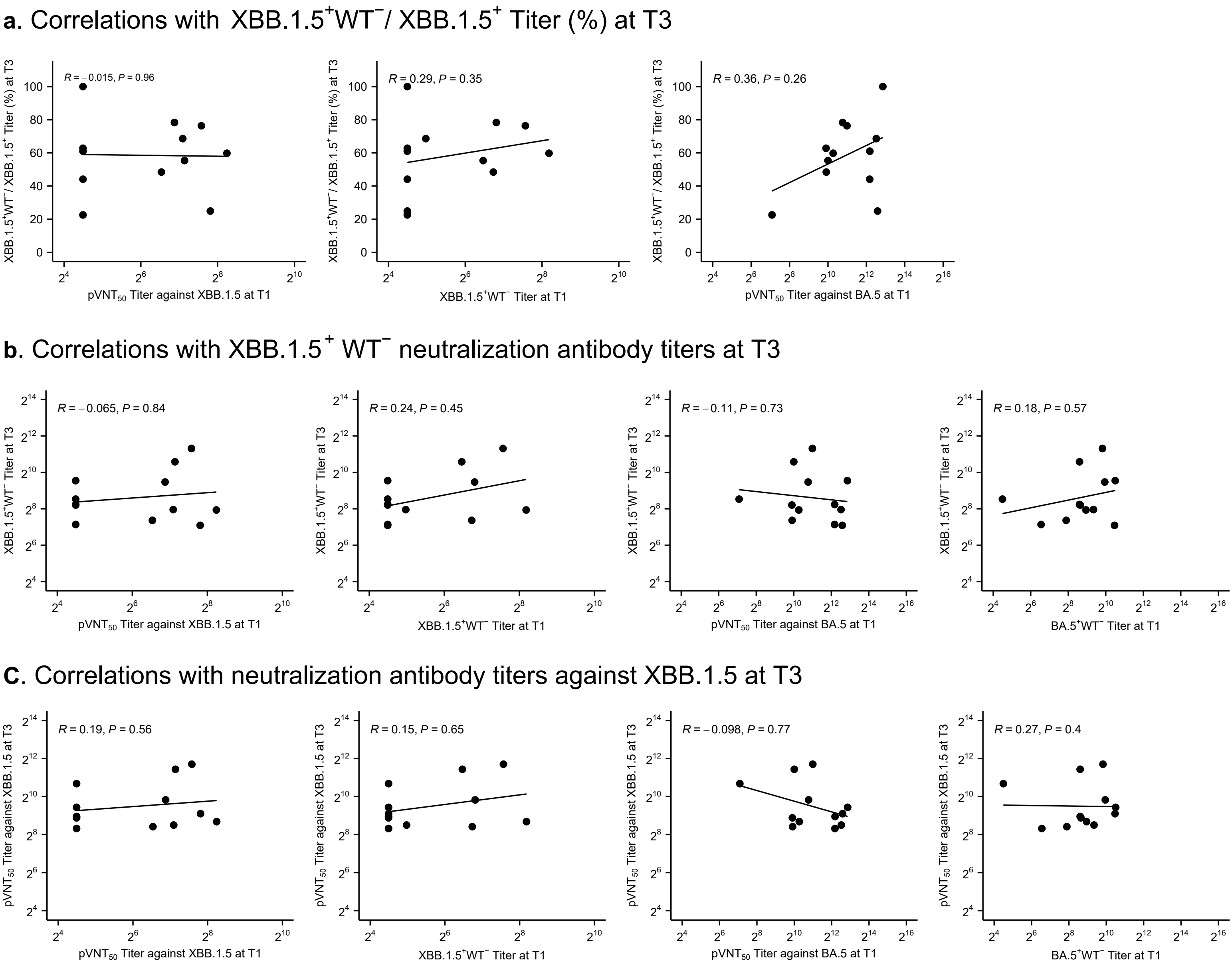

Supplementary Fig. S6 Correlations with XBB or BA.5 neutralization antibodies at T1 with XBB neutralization antibodies at T3.

(a) Correlations of XBB.1.5 and BA.5 neutralization antibody titers at T1 with the proportion of XBB.1.5 neutralization antibody that did not cross-react with WT at T3.

(b) Correlations of XBB.1.5 and BA.5 neutralization antibody titers at T1 with XBB.1.5 specific neutralization antibody titer that did not cross-react with WT at T3.

(c) Correlations of XBB.1.5 and BA.5 neutralization antibody titers at T1 with XBB.1.5 neutralization antibody titer at T3.

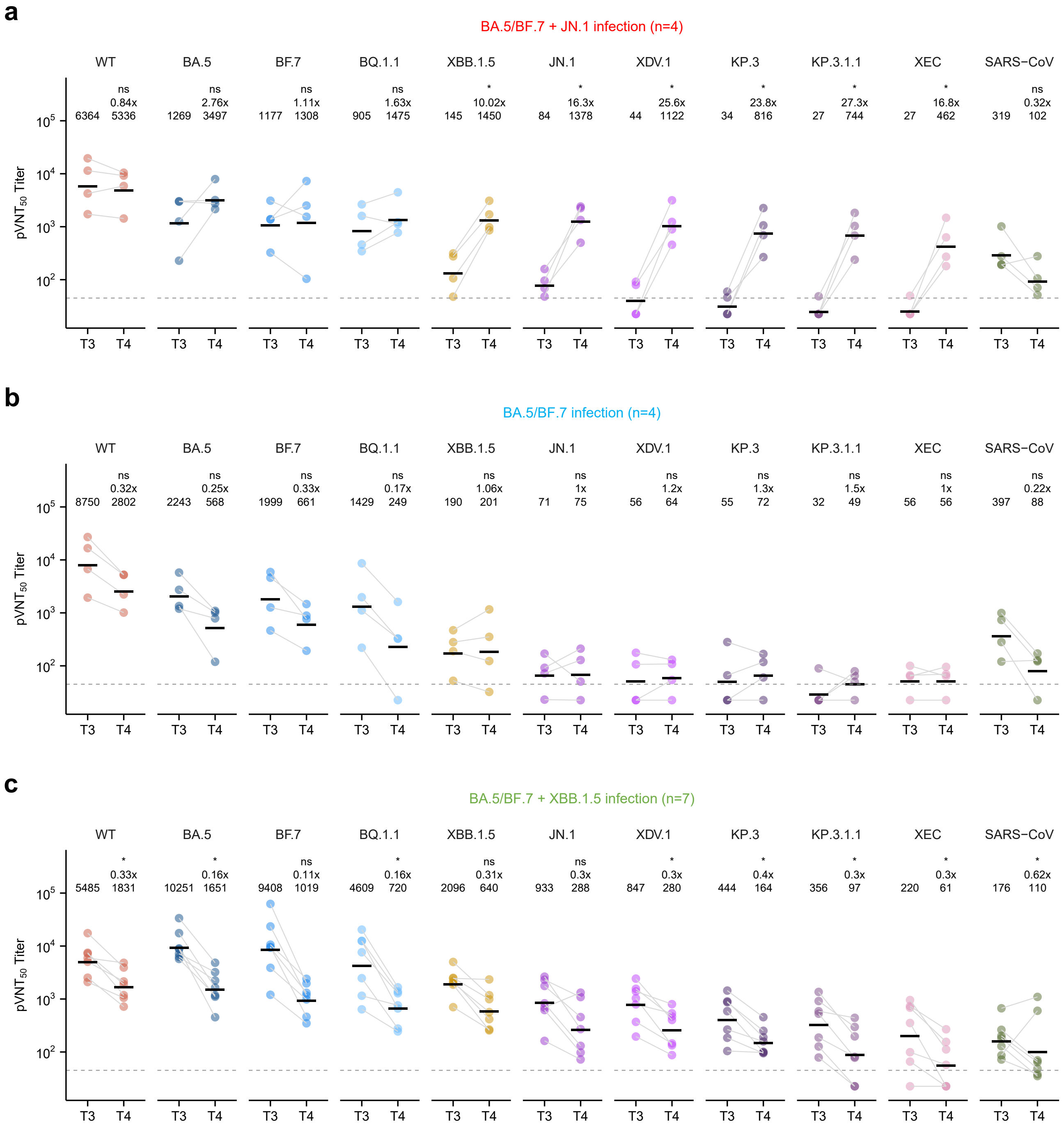

Supplementary Fig. S7 Dynamics of neutralization antibodies against SARS-CoV-2 variants at T3 and T4. Dashed lines indicate the limit of detection (pVNT50 = 45). Geometric mean titers (GMT) are labeled as black lines and shown above each column with fold-changes and significance compared with titer at T3 shown on the top. * *P* < 0.05, ** *P* < 0.01, *** *P* < 0.001; ns, not significant.

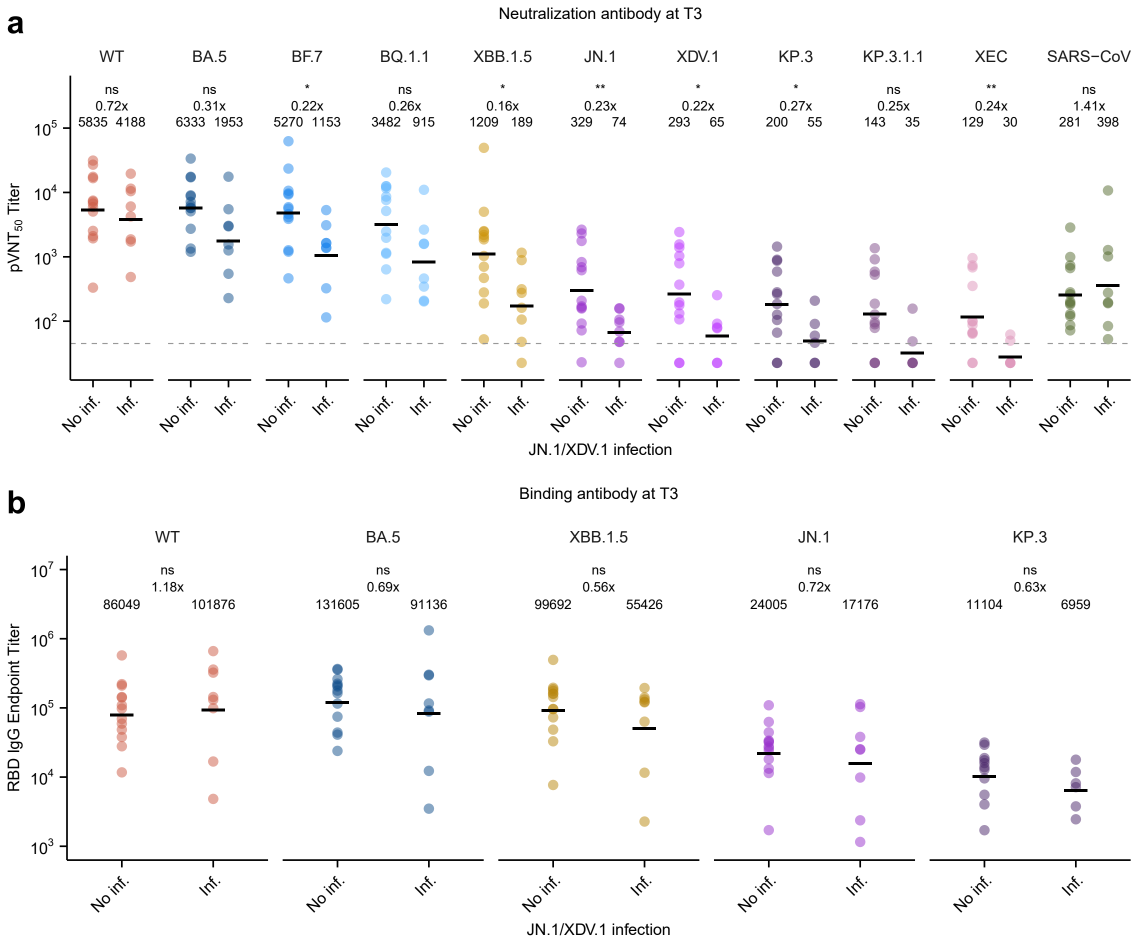

Supplementary Fig. S8 Comparisons of neutralizing antibody titers (a) and RBD-binding IgG titers (b) of the infected variants at T3 between individuals who were uninfected or infected with JN.1.

Supplementary Table S1 Demographic and vaccination history of participants.

Characteristic	Participants had BA.5/BF.7 (*n* = 10)	Participants had BA.5/BF.7 and XBB infection (*n* = 12)
Males (No., percent)	5 (50)	9 (64)
Age (Median, IQR)	30 (25.75, 33.5)	28 (25.75, 33.5)
Vaccination doses		
2	2	1
3	8	11
Serum samples		
T1	10	12
T2	5	10
T3	10	12

Supplementary Table S2 The demographic information and SARS-CoV-2 vaccination history of participants.

ID	Age	Gender	SARS-CoV-2 vaccination doses
1	32	M	3
2	29	F	3
3	28	F	3
4	29	F	2
5	32	F	3
6	27	M	0
7	26	M	1
8	23	M	3
9	27	F	2
10	26	M	3
11	33	F	3
12	33	M	3
13	46	M	3
14	27	M	3
15	31	F	1
16	32	M	3
17	32	M	2
18	24	F	3
19	59	M	2
20	29	F	3
21	24	F	3
22	26	F	3
23	25	F	3
24	25	F	3
25	25	M	3
26	34	F	3
27	28	F	3
28	35	F	3
29	32	F	3
30	25	M	3
31	33	M	3
32	28	F	3
33	33	M	3
34	23	F	3
35	24	F	3
36	28	M	3
37	26	M	3
38	27	F	3
39	35	M	3
40	54	F	2
41	23	F	3
42	30	M	0
43	36	F	3
44	26	F	3

45	27	M	3
46	22	F	3
47	22	F	3
48	32	F	3

Supplementary Table S3 Recombinant proteins used in this study.

Recombinant protein	Source	Product no.
SARS-CoV-2 Spike RBD protein	SinoBiological	40592-V08H
SARS-CoV-2 Omicron BA.5 Spike RBD protein	SinoBiological	40592-V08H130
SARS-CoV-2 Omicron XBB.1.5 Spike RBD protein	SinoBiological	40592-V08H146
SARS-CoV-2 Omicron JN.1 Spike RBD protein	SinoBiological	40592-V08H155
SARS-CoV-2 Omicron KP.3 Spike RBD protein	SinoBiological	40592-V08H157
SARS-CoV-2 Spike RBD protein, Biotinylated	SinoBiological	40592-V27H-B
SARS-CoV-2 Omicron BA.5 Spike RBD protein, Biotinylated	SinoBiological	40592-V49H9-B
SARS-CoV-2 Omicron XBB.1 Spike RBD protein, Biotinylated	SinoBiological	40592-V49H12-B
